# Therapeutic time window of multipotent adult progenitor therapy after traumatic brain injury

**DOI:** 10.1186/s12974-018-1122-8

**Published:** 2018-03-16

**Authors:** Supinder S. Bedi, Benjamin M. Aertker, George P. Liao, Henry W. Caplan, Deepa Bhattarai, Fanni Mandy, Franciska Mandy, Luis G. Fernandez, Pamela Zelnick, Matthew B. Mitchell, Walter Schiffer, Margaret Johnson, Emma Denson, Karthik Prabhakara, Hasen Xue, Philippa Smith, Karen Uray, Scott D. Olson, Robert W. Mays, Charles S. Cox

**Affiliations:** 1 Departments of Pediatric Surgery, University of Texas, Health Science Center at Houston, Houston, TX USA; 2 Departments of Surgery, University of Texas, Health Science Center at Houston, Houston, TX USA; 3 Michael E DeBakey Institute for Comparative Cardiovascular Science and Biomedical Devices and Athersys, Inc., Cleveland, OH USA; 4 Houston, USA

**Keywords:** Microglia, Neuroinflammation, Spatial learning and blood-brain barrier

## Abstract

**Background:**

Traumatic brain injury (TBI) is a major cause of death and disability. TBI results in a prolonged secondary central neuro-inflammatory response. Previously, we have demonstrated that multiple doses (2 and 24 h after TBI) of multipotent adult progenitor cells (MAPC) delivered intravenously preserve the blood-brain barrier (BBB), improve spatial learning, and decrease activated microglia/macrophages in the dentate gyrus of the hippocampus. In order to determine if there is an optimum treatment window to preserve the BBB, improve cognitive behavior, and attenuate the activated microglia/macrophages, we administered MAPC at various clinically relevant intervals.

**Methods:**

We administered two injections intravenously of MAPC treatment at hours 2 and 24 (2/24), 6 and 24 (6/24), 12 and 36 (12/36), or 36 and 72 (36/72) post cortical contusion injury (CCI) at a concentration of 10 million/kg. For BBB experiments, animals that received MAPC at 2/24, 6/24, and 12/36 were euthanized 72 h post injury. The 36/72 treated group was harvested at 96 h post injury.

**Results:**

Administration of MAPC resulted in a significant decrease in BBB permeability when administered at 2/24 h after TBI only. For behavior experiments, animals were harvested post behavior paradigm. There was a significant improvement in spatial learning (120 days post injury) when compared to cortical contusion injury (CCI) in groups when MAPC was administered at or before 24 h. In addition, there was a significant decrease in activated microglia/macrophages in the dentate gyrus of hippocampus of the treated group (2/24) only when compared to CCI.

**Conclusions:**

Intravenous injections of MAPC at or before 24 h after CCI resulted in improvement of the BBB, improved cognitive behavior, and attenuated activated microglia/macrophages in the dentate gyrus.

## Background

Traumatic brain injury (TBI) affects 1.5 million people in the USA annually, with 50,000 deaths due to acute injury [1]. Heretofore, interventions have been limited to modulating intracranial pressure and cerebral perfusion pressure, while long-term treatments consist of cognitive and behavioral rehabilitation [2]. Cellular therapy presents one of the most promising avenues to improve outcomes by limiting collateral inflammatory damage that follows the immediate trauma. Previously, we utilized cellular therapy to attenuate short-term (3–4 days after injury) and long-term effects (120 days after injury) of TBI in rodents [3–6]. Specifically, we have demonstrated that multiple doses of multipotent adult progenitor cells [MAPC (2/24 h after TBI)] at 10 million/kg body weight reduced blood-brain barrier (BBB) permeability and reduced the number of activated microglia/macrophages (based on morphology) in the dentate gyrus (no differences were found in the CA1 or CA3). These changes were accompanied by improvement in spatial learning in chronic animals (120 days) [5].

Multipotent adult progenitor cells are patented adult human-derived “off-the-shelf” cellular therapy. Multipotent adult progenitor cells are adherent cell type isolated from the bone marrow and other tissues [7], with their own transcriptome [8], secretome [9], miRNA profile [10, 11], and differentiation capability [8]. After injury, when MAPC are injected intravenously, they get trapped in the lungs and spleen rather than the brain [7]. Intravenous delivery of MAPC after TBI results in an increase in splenocyte and plasma T regulatory cell populations, with an increase in locoregional anti-inflammatory microglia/macrophages [12]. Multipotent adult progenitor cells treatment after cortical contusion injury (CCI) also resulted in a significant increase in anti-inflammatory cytokine interleukin-10 (IL-10) [3]. In vitro experiments demonstrated that direct contact between MAPC and splenocytes is required to modulate activated microglia [12]. In addition, in the absence of spleen, there is no effect of MAPC after TBI on microglia [3], thereby necessitating the involvement of spleen. Thus, these cells do not require honing to the brain to exert paracrine effect. Similar results have been demonstrated with other cell types in TBI [13].

Breakdown of the BBB following TBI results in increased vascular permeability, leading to cerebral edema. At the cellular level, neutrophils are the first to accumulate at about 24 h after injury [14]. Neutrophil accumulation is followed by leukocyte subsets that peak at about 3 days post injury [15]. Monocytes are recruited to the damaged brain in response to local chemokine signals, and once in the brain, they differentiate into macrophages [16]. Inflammatory monocytes are preferentially recruited to the injured brain [17]. Pro-inflammatory mediators peak at 24–36 h after injury [18, 19]. Infiltrating macrophages release pro-inflammatory cytokines such as interleukin-1 (IL-1) and tumor necrosis factor alpha (TNF-α) [18, 20–22]. The endogenous macrophages of the brain, microglia, are also activated and work to remove necrotic tissue and induce myelin repair [23, 24]. Resident microglia activation depends upon the biochemical milieu [25]. Microglia, in the presence of anti-inflammatory cytokines such as interleukin-4 (IL-4) and IL-10, are generally in a resting/ramified state [26]. In an unperturbed environment, microglia have a distinct morphology, consisting of a small, static cell body with dynamic and branched processes (inactivated). After a CNS injury, microglia are activated and transform into a distinct amoeboid-like morphology (activated). In stroke models, adult progenitor cell therapy leads to decreased tissue levels of pro-inflammatory cytokines such as interleukin-1 alpha (IL1α), interleukin-1 beta (IL1β), and TNF-α and interleukin-6 (IL-6) and increased levels of anti-inflammatory cytokine such as IL-10 [27, 28]. A recent clinical trial in ischemic stroke with MAPC demonstrated an advantage to treating at a time point less than 36 h post injury for MAPC treatment [29]. Similarly, in TBI, we hypothesized that there is an optimum clinically relevant time window of MAPC treatment in order for the therapy to attenuate the chronic harmful effects of TBI. To test this hypothesis, a series of experiments were completed to investigate the critical window of MAPC treatment to preserve the BBB, improve spatial learning, and attenuate activated microglia.

## Methods

All protocols involving the use of animals were in compliance with the National Institutes of Health Guide for the Care and Use of Laboratory Animals and were approved by the University of Texas Health Science Center Institutional Animal Care and Use Committee (AWC-11-120).

### Experimental design

Male rats at a starting average weight of 250 g were used in all experiments. Separate group of animals were used for the BBB studies and behavior testing. For BBB, we used four different time points for MAPC treatment. The time points were 2/24, 6/24, 12/36, and 36/72. Similarly, for the behavior testing at 120 days, we had the same MAPC treatment times (2/24, 6/24, 12/36, and 36/72). Animal experiments were performed in cohorts of 15–20 due to logistical consideration, and a positive control difference between sham and cortical contusion injury (CCI) was required in each cohort to be included to ensure the presence of a consistent injury. Animals were randomized to sham, CCI, or a MAPC dose following CCI. Numerous pre-clinical studies have demonstrated the feasibility of MAPC alone; thus, a sham + MAPC was not performed [13].

### Controlled cortical injury (CCI)

A controlled cortical impact device (Impact One Stereotaxic Impactor, Leica Microsystems, Buffalo Grove, IL) was used to administer a unilateral brain injury as described previously [30]. Adult male Sprague-Dawley rats weighing 250–300 g were anesthetized with 4% isoflurane and 0_2_, and the head was mounted in a stereotactic frame. The head was held in a horizontal plane, a midline incision was used for exposure, and a 6- to 7-mm craniectomy was performed on the right cranial vault. The center of the craniectomy was placed at the midpoint between bregma and lambda, ~ 3 mm lateral to the midline, overlying the tempoparietal cortex. Animals received a single impact of 2.7 mm depth (cognitive testing) or 3.1 mm depth (BBB) of deformation with an impact velocity of 5.6 m/s and a dwell time of 150 ms (moderate-severe injury) at an angle of 10° from the vertical plane using a 6-mm-diameter impactor tip, making the impact orthogonal to the surface of the cortex. The impact was made to the parietal association cortex. Sham injuries were performed by anesthetizing the animals, making the midline incision, and separating the skin, connective tissue, and aponeurosis from the cranium. The incision was then closed with sterile wound clips. A sham that includes a craniectomy can result in inflammation and anatomical damage that can confound results in a TBI model. Cole et al. demonstrated that a craniectomy resulted in injury that was distinct from the brain injury. In addition, there were changes in pro-inflammatory cytokines, morphological and behavioral damage as well [31]. Therefore, in order to avoid confounding results, we only did a midline incision as a sham.

### MAPC preparation and administration

MAPC were obtained from Athersys, Inc. (Cleveland, OH) and stored in liquid nitrogen. Prior to injection, the MAPC were thawed, washed, and suspended in phosphate-buffered saline (PBS) vehicle at a concentration of 10 × 10^6^ cells/kg in 1 ml of PBS (CCl-10). Cells were counted and checked for viability via Trypan blue exclusion. Viability was greater than 90%. Immediately prior to intravenous injection, MAPC (approximately 2.5 million in 1 ml) were titrated gently 8–10 times to ensure a homogeneous mixture of cells and were injected at variable time points after CCI injury via tail vein injection. CCI alone received PBS vehicle injection (1 ml) alone at the same designated time points as the cell-treated animals.

### Alexa Fluor 680 dye BBB permeability analysis

Animals that received MAPC at 2/24, 6/24, and 12/36 were euthanized 72 h post injury, and the 36/72 treated group was harvested at 96 h post injury. Prior to euthanasia, rats were anesthetized as described previously and 1 mg/kg of Alexa Fluor 680 dye conjugated to 10 kDa dextran PBS was introduced via tail vein injection [16]. The animals were allowed to recover for 30 min, then euthanized via right cardiac puncture and perfused with ice cold PBS followed by 4% paraformaldehyde. Explanted brains were placed in 4% paraformaldehyde for 1 h and then transferred to PBS. The brains were then sectioned coronally into 1-mm slices using a rat brain matrix slicer (Zivic Instruments, Pittsburgh, PA). Eight anatomically standardized slices encompassing the area of injury were placed on a plastic petri dish using a grid and imaged using an LI-COROdyssey CLx infrared laser scanner (LI-COR, Lincoln, NE) at 700 and 800 nm. Raw images were then stacked, processed, and analyzed in batch using Fiji, the fully open source version of ImageJ 1.48p (http://imagej.nih.gov/ij). Plot profiles were generated for each section along the impactor trajectory to identify the depth and anatomical region corresponding to the maximum Alexa Fluor signal intensity. The one-dimensional plot of Alexa Fluor signal intensity suggested that there may be two- to three-dimensional regions of BBB permeability after TBI corresponding to the injury penumbra. To identify potential regions of BBB permeability, threshold ranges were applied to the stacked raw images to detect various regional patterns of Alexa Fluor signal arising from the brain tissue from the site of cortical impact. Low + narrow signal intensity threshold ranges identified rims surrounding the area of maximal injury (4–5K, 5–7K, and 5–10K) whereas high + wide signal intensity threshold ranges identified the highest foci of signal within the most injured area of the cortex (5–60K). For each rat brain, the area of integrated signal from the Alexa Fluor was plotted against these intensity threshold ranges.

### Latency to platform

All behavior testing were done under blinded conditions, with unblinding after all the behavior testing was done. Cognitive function was tested using the Morris water maze (MWM) 120 days after injury to assess spatial memory and spatial learning. Animals were tested using four trials per day, over 6 consecutive days (during each week of testing). Each trial consisted of placing the animal in one of four starting locations (south, east, west, and north) chosen at random. The animal was gently placed in the tank facing the wall and allowed to search for the platform (located in north quadrant) for up to 60 s. If the animal failed to find the platform, it was placed upon the platform and allowed to remain for 30 s. Animal movement within the maze was monitored by a video camera linked to tracking software (Ethovision 3.1). Latency to platform was measured.

### Probe

To test memory retention, probe trials were administered 24 h after completion of the platform testing. Probe testing (60 s) involved removal of the platform and, using the tracking software, monitoring the animal movement. Calculations were then completed to determine the time the animal spent in the same quadrant as the platform (north quadrant) and time spent in the area three times the size of the platform (platform proximity duration). In addition, animal velocity was automatically calculated via the tracking software to evaluate for significant motor deficits as a result of injury.

### Tissue harvest

Following completion of all behavior testing, the animals were taken to the surgical suite where they were again anesthetized using isoflurane anesthesia. Under anesthesia, the animals’ chests were opened. Using a right ventrical puncture technique, the animals were simultaneously exsanguinated and perfused with 60 ml of ice cold PBS followed by 60 ml of ice cold 4% paraformaldehyde (PFA) at a rate of 20 cm^3^/min using a syringe pump. Following tissue fixation, the brains were removed and placed in 4% PFA and stored at 4° C.

### Immunohistochemistry

After harvest, the brains were transferred to a 30% sucrose solution, where they were maintained for at least 72 h. The brains were then put in a 3% agar mold and sectioned at 30 μm using a vibrating blade microtome (Leica Microsystems, Bannockburn, IL, USA). The sections were stained using a standard free-floating staining protocol. They were washed twice in PBS with 0.01% Triton X-100 [(PBST) T-8787, (Sigma Aldrich, St. Louis, MO, USA)] for 1 min and then incubated for 20–30 min in PBS with 0.2% Triton X-100. The sections were then blocked for 1 h at room temperature (RT) in 3% goat serum (# 005-000-121, Jackson Immune Research, PA) in PBST. A primary antibody was used to identify microglia/macrophages [Anti IBA1 Rabbit (1:100, Wake, Richmond, VA, USA)]. The antibody was prepared in PBTB [PBS with 0.01% Triton X-100, 2% bovine serum albumin (A9647, Sigma Aldrich, St. Louis, MO, USA)] and 1% goat serum and incubated at 4 °C overnight. The next day, the sections were rinsed briefly then washed with PBST and incubated with a secondary antibody [1:500; Goat anti-Rabbit IgG (H+L) Cross-Adsorbed, Alexa Fluor® 568:A 11011, lnvitrogen) in PBTB for 2 h at RT. The sections were again rinsed briefly and mounted, and cover-slipped with Fluoromount-G (Southern Biotechnology Associates, Birmingham, AL). Similar procedures were utilized to identify neurogenic cells [Anti Doublecortin Rabbit (DCX), AB55253, 1:1000 EMD Millipore, Billerica, MA, secondary antibody: 1:500; Goat anti-Rabbit IgG (H+L) Cross-Adsorbed, Alexa Fluor® 568:A 11011, lnvitrogen)].

### Quantification of immunohistochemistry

Immunohistochemistry was done after harvesting the brains post behavioral testing. Photomicrographs were taken of the hippocampus at × 200 using a Leica fluorescent microscope (Dm4000B LED). To maintain unbiased stereology [16], all image acquisition was performed by an investigator blinded to treatment of the individual histological sections. A single histological section per animal from mid-injury (interaural 5.70 mm, bregma − 3.30 mm) was examined. The dentate gyrus (DG) of the ipsilateral (right) and contralateral (left) hippocampus were photomicrographed (three random sections per slice) and quantified. Labeled cells were then classified based on morphology: small, static cell body with dynamic and branched processes was classified as inactivated, and cells that had an amoeboid morphology were classified as activated. A separate investigator, who was also blind to the group of each photomicrograph counted and characterized the phenotype of IBA1-positive cells. Images were unblinded after all the photomicrographs were analyzed. For the DCX-positive cells, the entire hippocampus was photomicrographed and counted. We photomicrographed and counted all groups.

### Statistical analysis

Unless otherwise indicated, all values are represented as mean ± SEM. Between group comparisons were analyzed using analysis of variance (ANOVA), and if found significant, they were further analyzed using Sidak’s multiple comparison test. A priori, we chose to use a built-in program in PRISM (GraphPad software) called ROUT (robust regression and outlier removal) and Grubbs to eliminate outliers for BBB and behavior testing. Additionally, animals were eliminated from analysis due to either death or procedural errors. Procedural errors included training in incorrect order in comparison to the rest of the animals in that group. *p* value of ≤ 0.05 was used to denote statistical significance. Statistical significance is indicated with “*” for *p* ≤ 0.05, “**” indicates statistical significance for *p* ≤ 0.01, “***” indicates statistical significance *p* ≤ 0.001, and “****” indicates statistical significance *p* ≤ 0.0001.

## Results

### MAPC treatment attenuates BBB permeability after TBI

**Fig. 1:**
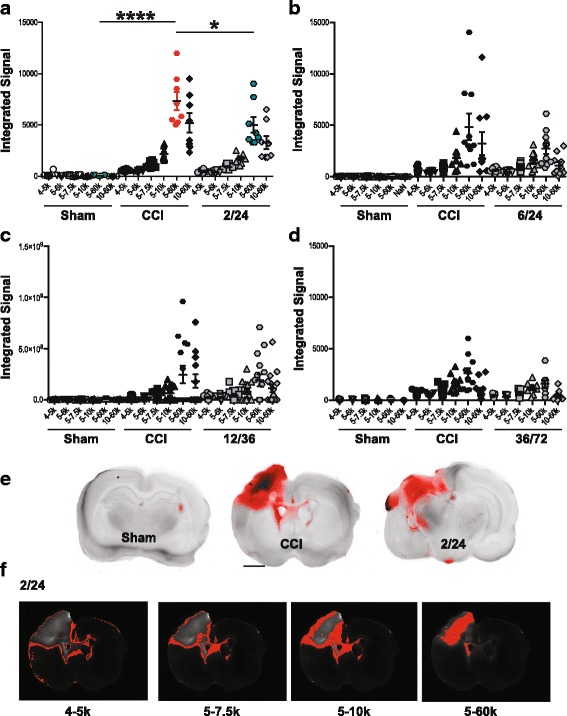
Intravenous multipotent adult progenitor cell treatment at 2/24 h after injury decreases brain permeability following traumatic brain injury. **a** A scatter plot representation of the integrated signal across each threshold intensity range for the three experimental groups for treatment at 2/24. **b** A scatter plot representation of the integrated signal across each threshold intensity range for the three experimental groups for treatment at 6/24. **c** A scatter plot representation of the integrated signal across each threshold intensity range for the three experimental groups for treatment at 12/36. **d** A scatter plot representation of the integrated signal across each threshold intensity range for the three experimental groups for treatment at 36/72. **e** Representative photomicrographs of 2-mm brain slices from rats that received MAPC treatment at 2/24 h after injury (CCI-2/24). Scale bar is 0.25 cm. **f** Representative images across each threshold intensity range for 2/24 treatment group. Abbreviations: CCI, cortical contusion injury (untreated) and CCI-2/24, cortical contusion injury treated with MAPC 2/24 h after injury

We utilized the integrated signal for regions using ranges of thresholds identified rim patterns corresponding to the penumbra and focal patterns corresponding to areas of maximal injury within the cortex. With multiple doses, we observed a significant reduction in the integrated signal between CCI (*n* = 8) and CCI-2/24 (*n* = 8) at 5–60K only (*p* < 0.05, Fig. 1a). There was also a significant difference between sham and CCI at 5–60K (*p* < 0.0001, Fig. 1a). There was a trend in reduction in the integrated signal between CCI and CCI-6/24 at 10–60K (*p* < 0.06, Fig. 1b), and there was a significant difference between sham and CCI at 10–60K (*p* < 0.01, Fig. 1a). In addition, we did not observe any differences in the integrated signal when animals were treated at 12/36 and 36/72 (Fig. 1c, d). There were a total of four data points that were considered outliers (two in 6/24 and two in the 36/72) that were eliminated from the data set of 600 data points (0.7% data eliminated).

### MAPC treatment at 2/24 and 6/24 reduces latency to hidden platform after TBI during training

**Fig. 2:**
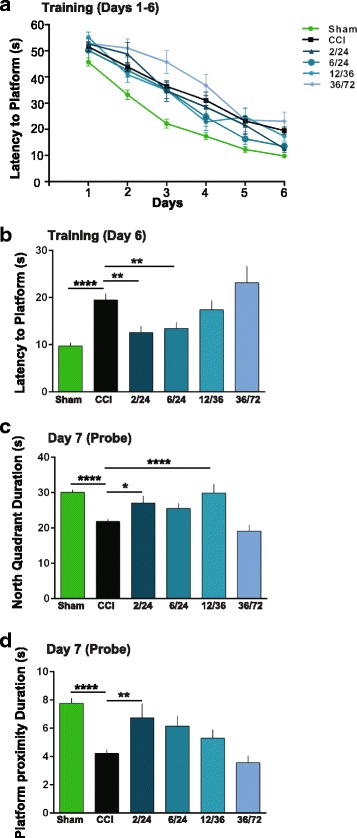
Intravenous multipotent adult progenitor cell treatment at 2/24 and 6/24 h after traumatic brain injury improved spatial learning and memory as measured by the attenuation in latency to find the hidden platform. **a** Six-day spatial training paradigm in Morris water maze. **b** Six-day comparison of latency among groups indicating significant decreases in latency at 2/24 and 6/24 with comparison with CCI. (**p ≤ .01) Abbreviations: CCI, cortical contusion injury (untreated); 2/24, cortical contusion injury treated with MAPC 2/24 h after injury; 6/24, cortical contusion injury treated with MAPC 6/24 h after injury; 12/36, cortical contusion injury treated with MAPC 12/36 h after injury and 36/72 cortical contusion injury treated with MAPC 36/72 h after injury. **c** Duration in north quadrant increased significantly when MAPC was administered at 2/24 (*p ≤ .05) and 12/36 (***p ≤ .001) hours after injury. **d** Duration spent in platform proximity increased significantly when MAPC was administered at 2/24 (**p ≤ .01) and 6/24 (*p ≤ .05) hours after injury. Abbreviations: CCI, cortical contusion injury (untreated); 2/24, cortical contusion injury treated with MAPC 2/24 h after injury; 6/24, cortical contusion injury treated with MAPC 6/24 h after injury; 12/36, cortical contusion injury treated with MAPC 12/36 h after injury and 36/72 cortical contusion injury treated with MAPC 36/72 h after injury

In order to measure spatial learning, we used the Morris water maze (MWM) spatial learning paradigm. Spatial learning was conducted over a 6-day period 120 days after TBI (Fig. 2a) followed by a probe trial on day 7 (see the “Methods” section). Repeated measures ANOVA *p* value was < 0.001.

There was a significant difference in latency to platform on day 6 between CCI (*n* = 65, 19.5 ± 1.5) and sham (*n* = 55, 9.8 ± 0.7; *p* < 0.0001, Fig. 2b). Dosing at 2/24 resulted in significant reduction in latency to platform between CCI and CCI-2/24 (*n* = 13, 12.6 ± 1.4, *p* ≤ 0.01, Fig. 2b). In addition, we observed significant differences in attenuation of latency to platform between CCI and CCI-6/24 (*n* = 14, 13.5 ± 1.3, ≤ 0.01, Fig. 2b). There was no difference in latency to platform between CCI and CCI-12/36 (*n* = 15, 17.4 ± 1.9, *p* > 0.05, Fig. 2b). Animals that received MAPC at 36/72 h after injury displayed an increase in latency to platform between CCI and CCI-36/72 (*n* = 14, 23.1 ± 3.5, *p* > 0.05, Fig. 2b). Additionally, we did not see any significant differences in swim speeds across all groups (data not shown), confirming that there were no systemic limitations to account for any observed differences in latency times. There was one outlier in the 2/24 and two in 6/24. Additionally, there was one death in 36/72 and seven procedural errors in 12/36.

### MAPC treatment at 2/24 and 12/36 increases duration in the north quadrant after TBI during probe trial

Overall, there was significant difference in the time spent in the north quadrant during the probe trial comparing CCI (*n* = 65, 21.7 ± 0.8) and sham (*n* = 55, 30.1 ± 0.8, *p* < 0.0001, Fig. 2c). Animals treated with MAPC at 2/24 h post injury demonstrated a significant increase in duration in the north quadrant between CCI and CCI-2/24 (*n* = 13, 27.1 ± 2.1, *p* < 0.01, Fig. 2c). The animals that received treatment at 6/24 h after injury spent more time in north quadrant, but the difference was not significant between CCI and CCI-6/24 (*n* = 14, 25.5 ± 1.5 *p* > 0.05, Fig. 2c). Interestingly, there was a significant increase in duration in the north quadrant between CCI and CCI-12/36 (*n* = 15, 29.9 ± 2.5, *p* < 0.0001, Fig. 2c). Similar to latency, there was no difference in duration in the north quadrant between CCI and between CCI and CCI-36/72 (*n* = 14, 19.1 ± 1.8, *p* > 0.05, Fig. 2c).

### MAPC treatment at 2/24 and 6/24 increases platform proximity duration after TBI

Time spent near the platform was significantly different (CCI *n* = 65, 4.2 ± 0.3) during the probe trial vs sham (*n* = 55, 7.8 ± 0.4, *p* < 0.0001, Fig. 2d). With the double-dosing strategy, there was a significant increase in platform proximity duration between CCI and CCI-2/24 (*n* = 13, 6.7 ± 1.0, *p* < 0.01, Fig. 2d). There was no significant increase at CCI-6/24 (*n* = 14, 6.1 ± 0.7, *p* > 0.05, Fig. 2d) from CCI. With treatment at the 12/36 time point, a non-significant increase in platform proximity from CCI was observed (CCI-12/36 *n* = 15, 5.3 ± 0.6, *p* > 0.05, Fig. 2d). There was no difference in platform proximity duration between CCI and CCI-36/72 (*n* = 14, 3.6 ± 0.5, *p* > 0.05, Fig. 2d).

### MAPC treatment at 2/24 h reduces the number of activated microglia/macrophages in the dentate gyrus

**Fig. 3:**
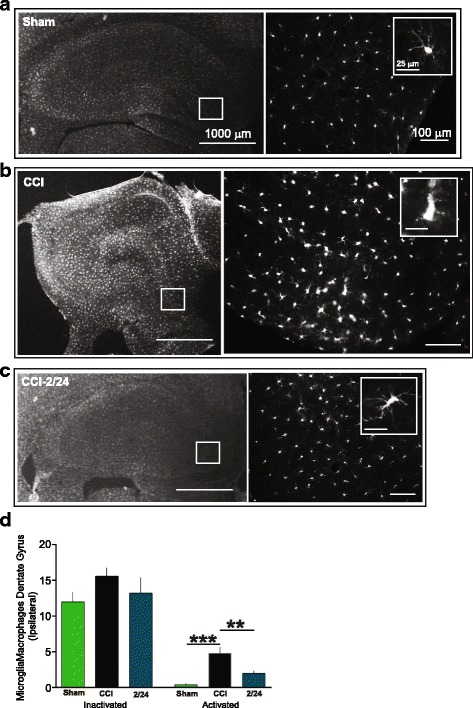
Multipotent adult progenitor cell treatment reduced the number of activated microglia/macrophages in the ipsilateral dentate gyrus. Traumatic brain injury resulted in chronic activation of microglia/macrophages that was attenuated with MAPC treatment at 2/24 h after injury in the dentate gyrus. **a**–**c** Photomicrographs of microglia/macrophages from sham (**a**), CCI (**b**), and CCI-2/24 (**c**) animals using IBA1 antibody (white). Scale bars 1000 μm (× 1.25), 100 μm (× 20); inset 25 μm. **d** Number of inactivated and activated microglia/macrophages in the dentate gyrus of the ipsilateral hippocampus at 2/24 h

**Fig. 4:**
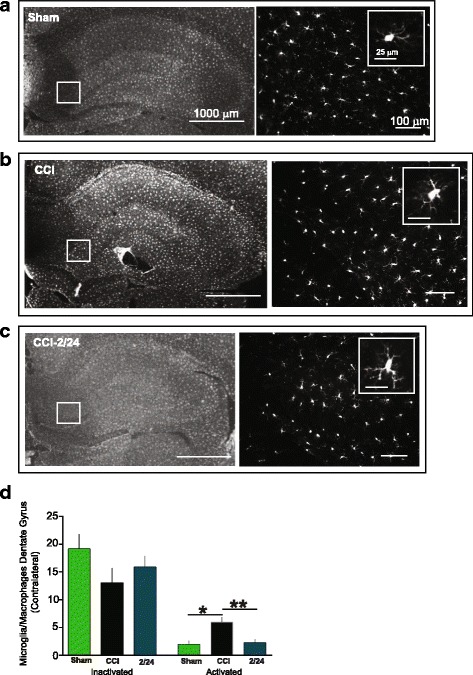
Multipotent adult progenitor cell treatment reduced the number of activated microglia/macrophages in the contralateral dentate gyrus. Traumatic brain injury resulted in chronic activation of microglia/macrophages that was attenuated with MAPC treatment at 2/24 h after injury in the dentate gyrus. **a**–**c** Photomicrographs of microglia/macrophages from sham (**a**), CCI (**b**), and 2/24 (**c**) animals using IBA1 antibody (white). Scale bars 1000 μm (× 1.25), 100 μm (× 20); inset 25 μm. **d** Number of inactivated and activated microglia/macrophages in the dentate gyrus of the contralateral hippocampus

**Fig. 5:**
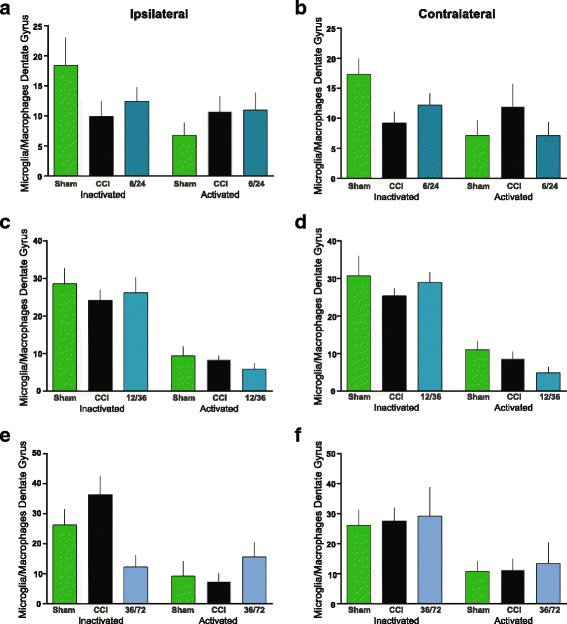
Multipotent adult progenitor cell treatment has no effect on microglia/macrophages when administered at 6/24, 12/36, or 36/72 h after injury. **a** 6/24 MAPC treatment: ipsilateral microglia/macrophages count based on morphology. **b** 6/24 MAPC treatment: contralateral microglia/macrophages count based on morphology. **c** 12/36 MAPC treatment: ipsilateral microglia/macrophages count based on morphology. **d** 12/36 MAPC treatment: contralateral microglia/macrophages count based on morphology. **e** 36/72 MAPC treatment: ipsilateral microglia/macrophages count based on morphology. **f** 36/72 MAPC treatment: contralateral microglia/macrophages count based on morphology

We counted the number of activated and inactivated microglia/macrophages in the dentate gyrus in the ipsilateral and contralateral hippocampus in all groups. CCI-2/24 (2.0 + 0.4) had significantly (*p* < 0.05) fewer activated microglia/macrophages in the DG than CCI (*n* = 10, 4.8 + 0.9) in the ipsilateral hippocampus (Fig. 3d). There was a significant difference (*p* < 0.01) between CCI and sham (*n* = 5, 0.4 + 0.2). Similar significant (*p* < 0.01) changes were observed in the contralateral hippocampus with CCI-2/24 (2.3 + 0.8) vs CCI (6.0 + 0.9, Fig. 4d). There was a significant difference (*p* < 0.01) between CCI and sham (*n* = 5, 0.4 + 0.7, Fig. 4d). There were no significant differences in the other dosing times (Fig. 5). In addition, there were no differences in inactivated microglia/macrophages among all groups (Figs. 3, 4, and 5).

### MAPC treatment does not affect chronic neurogenesis

**Fig. 6:**
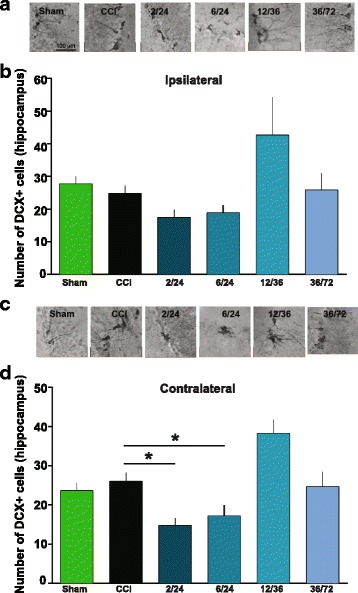
Multipotent adult progenitor cell treatment does not affect neurogenesis as measured by DCX+ cells in the hippocampus. **a** Ipsilateral: photomicrographs of DCX+ cells in the hippocampus in the various groups [100 μm (× 20)]. **b** Ipsilateral: DCX+ cells in the various groups. There were no significant differences in the number of DCX+ cells when compared to CCI. **c** Contralateral: photomicrographs of DCX+ cells in the hippocampus in the various groups. **d** Contralateral: DCX+ cells the various groups. There were significant reductions in the number of DCX+ cells in MAPC-treated groups at 2/24 and 6/24 when compared to CCI

We counted all the DCX-positive cells in the ipsilateral and contralateral hippocampus. Fewer DCX-positive cells were observed in the ipsilateral hippocampus (Fig. 5a) of animals treated at 2/24 (*n* = 10, 17.5 ± 2.4) and 6/24 (*n* = 11, 18.9 ± 2.3) when compared to CCI animals (*n* = 55, 24.8 ± 2.3). There was no difference between CCI and sham (*n* = 45, 27.8 + 2.3) (Fig. 6). Interestingly, in the contralateral hippocampi (Fig. 6b), there was a significant reduction in DCX-positive cells in groups that were treated with MAPC at 2/24 (*n* = 10, 14.8 + 1.9, *p* < 0.05) and 6/24 (*n* = 12, 17.2 + 2.7, *p* < 0.05) when compared to CCI (*n* = 56, 26.1 + 2.1). There was an increase in neurogenesis at 12/36 (*n* = 7, 38.3 + 3.5) when compared to CCI. There was no difference between CCI and sham (*n* = 47, 23.7 + 2.0).

## Discussion

Our data provide evidence that there is a critical window of 24–36 h post injury in which MAPC treatment reduces BBB permeability, improves cognitive behavior, and decreases activated microglia/macrophages, and these changes are independent of neurogenesis. These data parallel the recently completed ischemic stroke phase II clinical trial and should guide clinical testing paradigms. Furthermore, this study replicates previous results in two species [3–5], adding robustness to the observations.

TBI results in the disruption of the BBB, which allows for an increase in vascular permeability [3]. In the absence of macrophages from the periphery, there is a reduction in microglial activation [32]. Microglia, the resident macrophages of the brain, when activated following injury, remove necrotic tissue and induce myelin repair [33]. Thus, by clearing out the dead cells and debris, activated microglia may serve a neuroprotective role. However, in many neurological diseases, microglia can have sustained secretion of inflammatory cytokines and cytotoxic molecules—such as IL-1, TNF-alpha, and nitric oxide—compounded with excessive neuronophagia, can have deleterious effects [18, 33] . MAPC attenuate BBB permeability by directly interacting with the spleen to alter the systemic immunologic/inflammatory response [3, 4]. Subsequently, pro-inflammatory microglia polarize into an anti-inflammatory phenotype. Though the precise mechanism by which this happens is yet unknown, splenocytes were shown to release anti-inflammatory cytokines such IL-10, IL-4, and others [12]. By reducing BBB permeability, there is a likely reduction of infiltrating peripheral macrophages, and therefore, a reduction in microglial activation, and conversion of pro-inflammatory microglia into anti-inflammatory microglia. After a CNS injury, pro-inflammatory mediators such as neutrophils and cytokines peak at 24–36 h post injury followed by anti-inflammatory cytokines for 96 h post injury [14, 18, 34]. We observed a significant decrease in BBB permeability using Alexa Fluor 680 [35], when MAPC were administered at 2/24 h (Fig. 1a), but not post 24 h after injury. Surprisingly, MAPC treatment at 6/24 did not significantly attenuate BBB, though there was a trend in reduction of BBB at 10–60K (*p* = 0.06). Treatment with MAPC after 24 h was ineffective in attenuating BBB permeability.

Similar to the BBB data, our cognitive behavioral data also support a critical time window of MAPC treatment of 24 h post injury. We found an improvement in spatial learning as measured by the Morris water maze if MAPC were administered prior to 24 h (Fig. 2). Spatial learning heavily relies on the circuitry of the hippocampus [36]. Acquisition and spatial localization of relevant visual cues are consolidated, retained, and recalled over the training and probe trials [37]. The dentate gyrus is a critical locus in the hippocampus that is involved in the acquisition of spatial memory [38, 39]. Lesions of the hippocampus, dentate gyrus, and ventral subiculum result in poor post training probe trials [38, 40]. In our experiments, we observed improvement in the latency on day 6 of training, when MAPC were administered 24 h post injury. Specifically, we saw improvement with doses administered at 2/24 and 6/24 h after injury on training day 6 (Fig. 2b). These results are similar to our previously observed changes at 2/24 [5]. We observed moderate improvements in latencies when cells were administered at 12/36 and no change when cells were administered at 36/72 h after injury. Both (12/36 and 36/72) were not significantly different than CCI (Fig. 2b). In addition, we observed significant improvement in the recall of the hidden platform during the probe trials. In the probe trial, when the hidden platform is removed, animals that received doses at or prior to 24 h after injury (2/24 and 12/36) demonstrated a spatial bias to the quadrant of the former location of platform (Fig. 2c). Spatial bias to the former location was also observed with platform proximity duration. Significant increases in platform proximity duration were observed in animals that received MAPC treatment at 2/24 only (Fig. 2d). Both, north quadrant duration and platform proximity duration, indicated that MAPC treatment given prior to 24 h is most effective in improving spatial memory. MAPC treatment given at 36/72 h after injury had worse outcomes than CCI (Fig. 2). Recent data from phase II clinical trial in ischemic stroke patients treated with MAPC also demonstrated similar results. Patients given MAPC 36 h after stroke were no different than patients given placebo. Patients that were given MAPC at 24 h showed significant clinical improvement over patients given placebo [29]. Our preclinical data parallels the phase II clinical data (Fig. 2).

Neuronal function and subsequent behavior can be affected negatively by neuroinflammation, which in part is mediated by activated microglia [41]. Case analyses of TBI patients have shown presence of activated microglia with white matter degeneration ranging from months up to 47 years [42, 43]. Activation states of microglia can be categorized by morphological features and/or expression of surface or intracellular markers, as previously described [23, 25, 44]. Twenty-four to 120 h after TBI, MAPC treatment alters the ratio of pro-inflammatory (CD86+: activated) microglia/macrophages to anti-inflammatory (CD206+: inactivated) microglia/macrophages in favor of an inactivated state [12]. In the absence of treatment after injury, we observe an increase in pro-inflammatory (activated) vs anti-inflammatory (inactivated) microglia/macrophages [45]. Using IBA1 to identify microglia/macrophages and subsequently categorizing them based on morphology, we observed a decrease in activated microglia/macrophages in the dentate gyrus ipsilateral to the injury when given doses at 2/24 h after injury when measured at a chronic time point of 120 days after injury [5].

Similarly, in our current experiments, we observed a significant decrease in activated microglia/macrophages in the dentate gyri ipsilateral to the injury when MAPC were given at 2/24 (Fig. 3). Interestingly, we found a significant decrease in activated microglia/macrophages in the contralateral dentate gyri as well (Fig. 4). However, there were no significant changes in the inactivated phenotype of microglia/macrophage at 2/24. There were decreases in activated microglia/macrophages with two doses (6/24 and 12/36) when compared to CCI, but these changes were not significant (Fig. 5a–d). When animals were treated with MAPC at 36/72 h after the injury, there was an increase in activated microglia/macrophages (Fig. 5e, f). These results indicate that MAPC treatment is effective at reducing activated microglia/macrophages when administered 2/24 h after injury, but when administered 36/72 h after injury, there is an increase in activated microglia/macrophages. Future studies will correlate pro- and anti-inflammatory surface markers with activated/inactivated microglial/macrophage morphology. Since neuronal function and behavior can be negatively affected by neuroinflammation [41], a reduction in activated microglia/macrophages might indeed contribute to the improvement in spatial learning observed in the Morris water maze (Fig. 2). Another possibility for the improvement is spatial learning after MAPC treatment might be due to neurogenesis. Mammalian neurogenesis can be triggered due to a proliferation of endogenous neural stem cells [46] which can replace populations of damaged neurons. Recent evidence by Kota et al. demonstrated an increase in neurogenesis with cellular therapy in the short term only (7 days after injury) [47]. However, we observed an increase in neurogenesis in the contralateral hippocampus at 12/36 only in which we observed changes in the time spent in the north quadrant (Fig. 2c). Alternatively, there were significant reductions of DCX-positive cells in the contralateral hippocampi at 2/24 and 6/24. Perhaps, MAPC treatment does not affect neurogenesis. It is also possible that neurogenesis occurs acutely after injury [48] and measured at 4 months post injury in this experiment does not capture those findings.

## Conclusion

Intravenous injections of MAPC at or before 24 h resulted in improvement of the BBB, improved cognitive behavior, and attenuated activated microglia/macrophages in the dentate gyrus. In addition, neurogenesis does not play a role in the improved behavior with MAPC treatment after injury. These data will be important in guiding future clinical dosing strategies for treating TBI with MAPC.

## Abbreviations

ANOVA: Analysis of variance
BBB: Blood-brain barrier
CCI: Cortical contusion injury
DCX: Anti Doublecortin Rabbit
DG: Dentate gyrus
IBA1: Ionized calcium-binding adaptor molecule 1
IL-1: Interleukin-1
IL-10: Interleukin-10
IL1α.: Interleukin-1 alpha
IL1β: Interleukin-1 beta
IL-4: Interleukin-4
MAPC: Multipotent adult progenitor cells
MWM: Morris water maze
PBS: Phosphate-buffered saline
PFA: Paraformaldehyde
TBI: Traumatic brain injury
TNF-α: Tumor necrosis factor alpha
